# Supplementary material for: Phage-specific immunity impairs efficacy of bacteriophage targeting Vancomycin Resistant Enterococcus in a murine model
Source: Nat Commun. 2024 Apr 6;15:2993. doi: 10.1038/s41467-024-47192-w (PMC10998888; doi:10.1038/s41467-024-47192-w)
Supplement: Supplementary file 1 — Supplementary Information [file 41467_2024_47192_MOESM1_ESM.pdf]

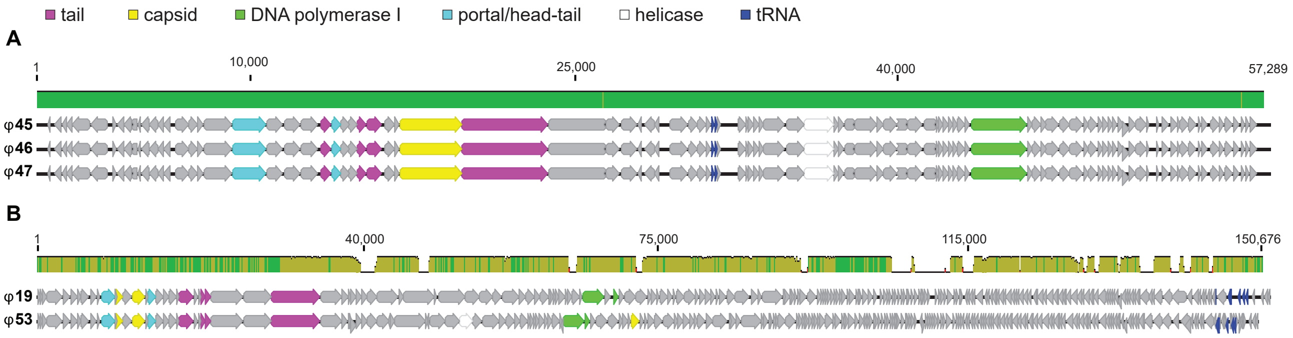

Supplement Figure 1. Whole genome sequencing of phages targeting VRE

Whole genome sequencing and annotation is shown for phages in the cocktail with major protein families labeled. (A) Alignment of siphophages ϕ45, ϕ46, and ϕ47 (B) alignment of myophages phages ϕ19 and ϕ53. Mean pairwise identity over all pairs in the column. Green represents 100% identity. Yellow represents at least 30% and under 100% identity. Red represents below 30% identity.

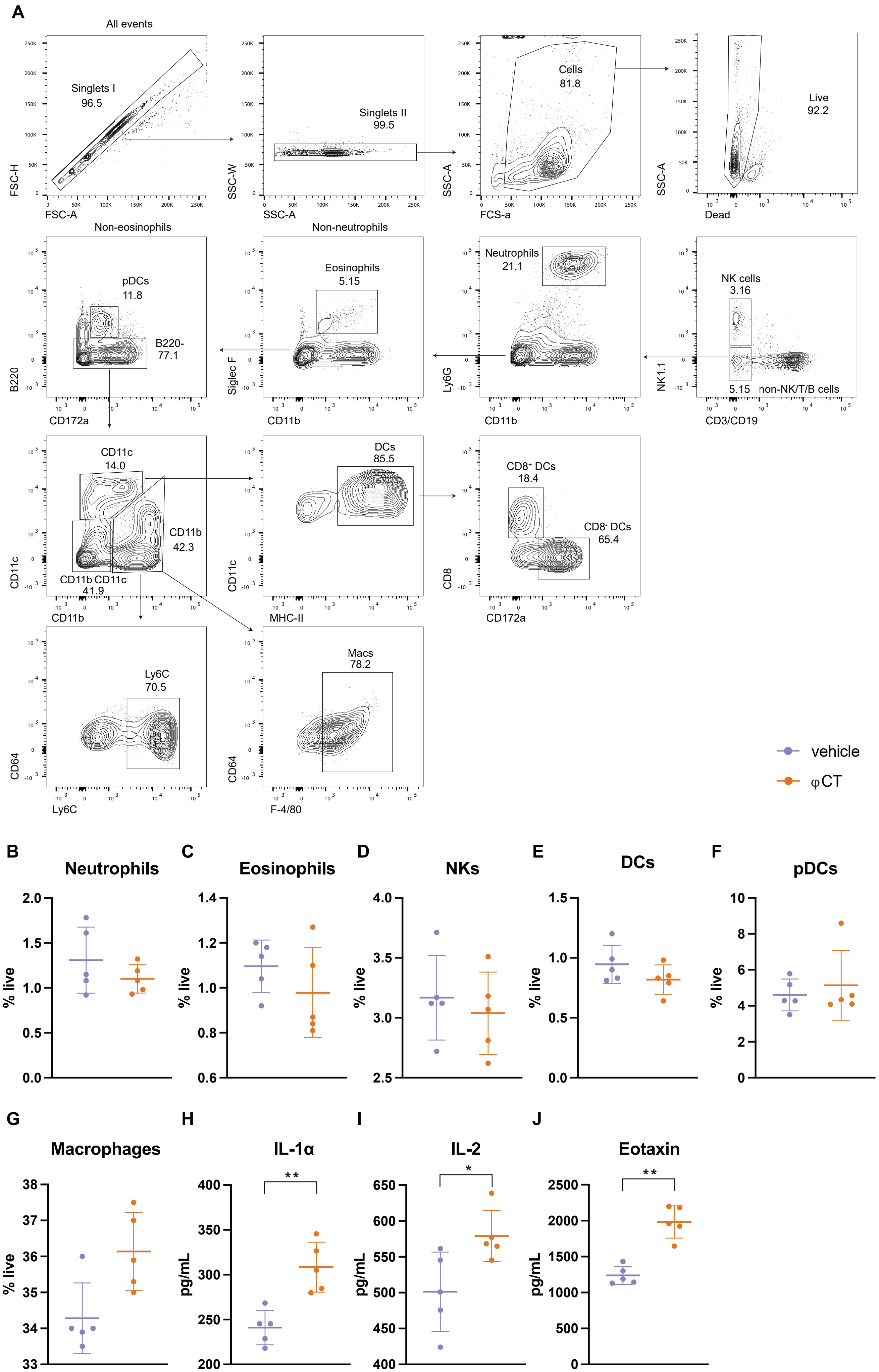

A
All events
Singlets I
96.5
FSC-H
FSC-A
Singlets II
99.5
SSC-W
SSC-A
Cells
81.8
SSC-A
FCS-a
Live
92.2
SSC-A
Dead
Non-eosinophils
pDCs
11.8
B220-
77.1
B220
CD172a
Non-neutrophils
Eosinophils
5.15
Siglec F
CD11b
Neutrophils
21.1
Ly6G
CD11b
NK cells
3.16
5.15
non-NK/T/B cells
NK1.1
CD3/CD19
CD11c
14.0
CD11b
42.3
CD11b-CD11c-
41.9
CD11c
CD11b
DCs
85.5
CD11c
MHC-II
CD8+ DCs
18.4
CD8- DCs
65.4
CD8
CD172a
Ly6C
70.5
CD64
Ly6C
Macs
78.2
CD64
F-4/80
vehicle
φCT
B
Neutrophils
% live
C
Eosinophils
% live
D
NKs
% live
E
DCs
% live
F
pDCs
% live
G
Macrophages
% live
H
IL-1α
pg/mL
**
I
IL-2
pg/mL
*
J
Eotaxin
pg/mL
**

Supplement Figure 2. Innate immunity to phage cocktail

Mice were administered daily IP injections with either the ϕCT or vehicle control for seven days. One day after the last injection (day 7), spleens were harvested for flow cytometry and the Bio-plex system. (A) Gating strategy to identify innate immune cell populations. (B-G) Frequencies of innate cell populations in the spleen. (H-J) Cytokine production in the spleen. Only cytokines that were significantly different between treatment groups are shown. * $p < 0.01$, ** $p < 0.001$ by Student's t test. Data are representative of two experiments with n=5 per group.

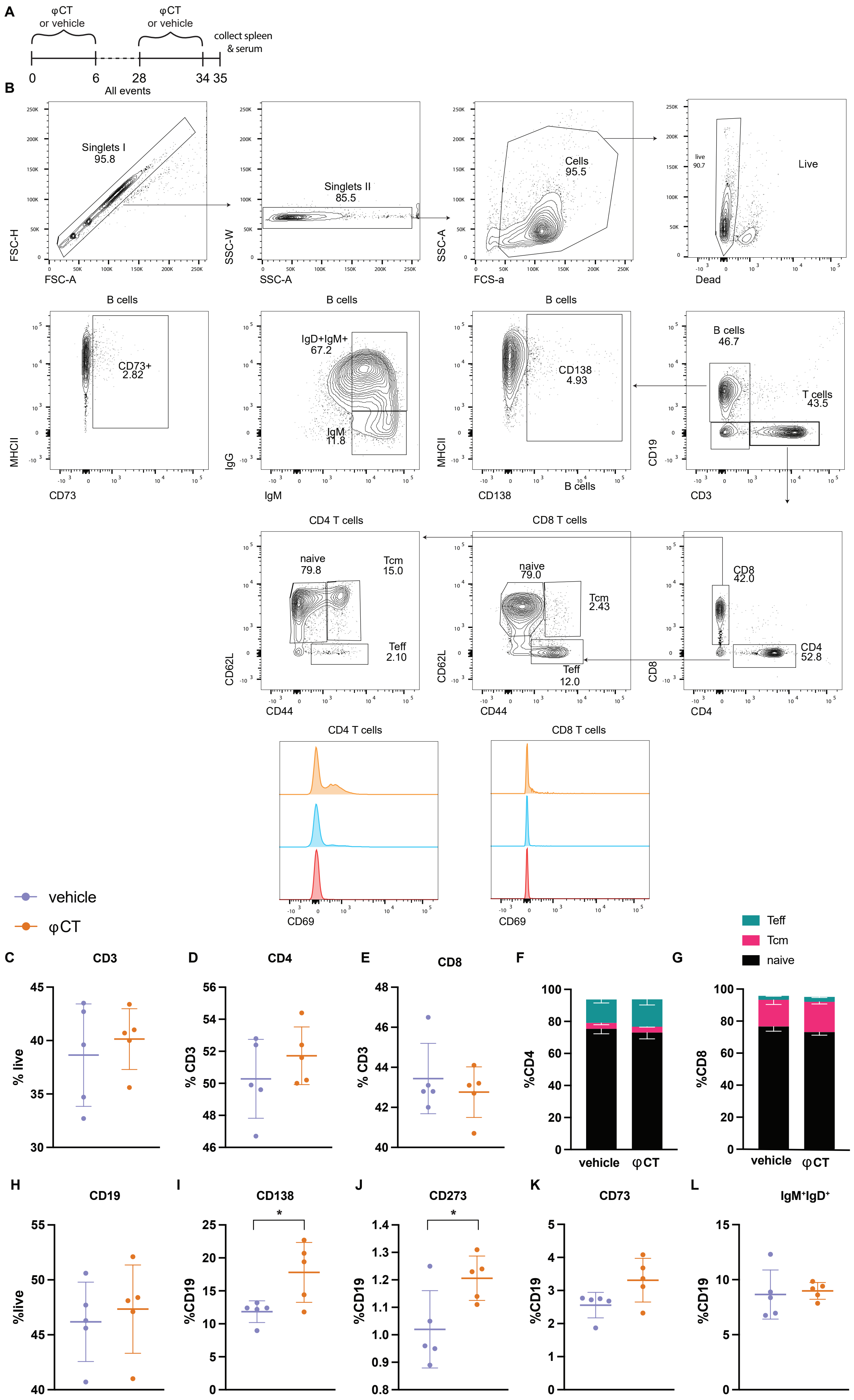
A
φCT or vehicle
φCT or vehicle
collect spleen & serum
0
6
28
34 35
B
All events
Singlets I 95.8
FSC-H
FSC-A
Singlets II 85.5
SSC-W
SSC-A
Cells 95.5
SSC-A
FCS-a
live 90.7
Live
Dead
B cells
CD73+ 2.82
MHCII
CD73
B cells
IgD+IgM+ 67.2
IgM 11.8
IgG
IgM
B cells
CD138 4.93
MHCII
CD138
B cells 46.7
T cells 43.5
CD19
CD3
CD4 T cells
naive 79.8
Tcm 15.0
Teff 2.10
CD62L
CD44
CD8 T cells
naive 79.0
Tcm 2.43
Teff 12.0
CD62L
CD44
CD8 42.0
CD4 52.8
CD8
CD4
CD4 T cells
CD69
CD8 T cells
CD69
vehicle
φCT
Teff
Tcm
naive
C CD3 % live
D CD4 % CD3
E CD8 % CD3
F %CD4 vehicle φCT
G %CD8 vehicle φCT
H CD19 %live
I CD138 %CD19 *
J CD273 %CD19 *
K CD73 %CD19
L IgM+IgD+ %CD19

Supplement Figure 3. Phenotyping of adaptive immune cells after two treatments of phage therapy

(A) Mice were treated with either ϕCT or vehicle control for seven days and then one month later, treated with the same regime. Spleens were harvested one day after secondary treatment (day 35) for flow cytometry analysis. (B) Gating strategy to identify adaptive immune populations. Frequencies and phenotyping of (C-G) T cells and B cells (H-L). Groups were compared by student's T test. Data are representative of two experiments with n=5 per group.

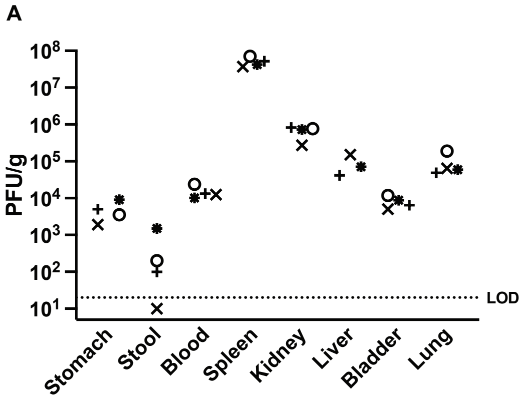

Supplement Figure 4. Phage biodistribution in the tissues

Mice were administered one dose of ϕCT via IP injection. After 24 hours, organs were harvested and plated to detect phage using plate titer. Different shapes depict individual mice. One stool sample (cross) was below the limit of detection and one liver sample (circle) was lost during processing. This experiment is representative of two replicates with n=4 mice per group.

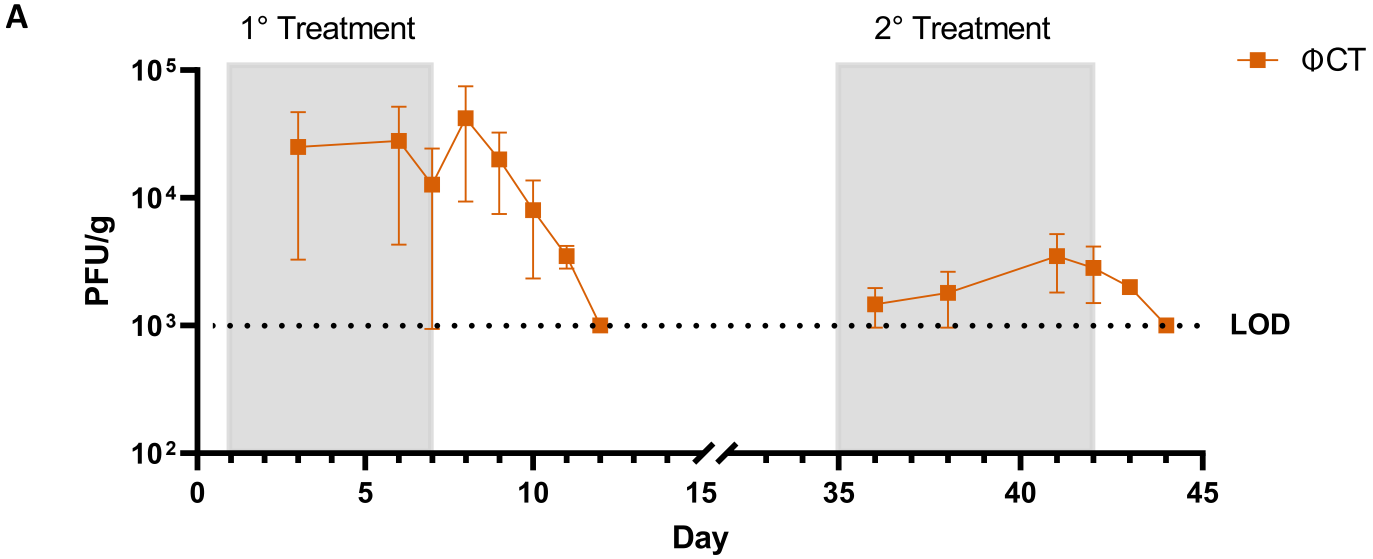

Supplement Figure 5. Characterization of phage and VRE after two courses of phage cocktail or vehicle control. Mice were administered two doses of phage cocktail, 4 weeks apart, via IP injection. Stool was collected and enumerated for phage using plate titer. 10 mice were included in this experiment.

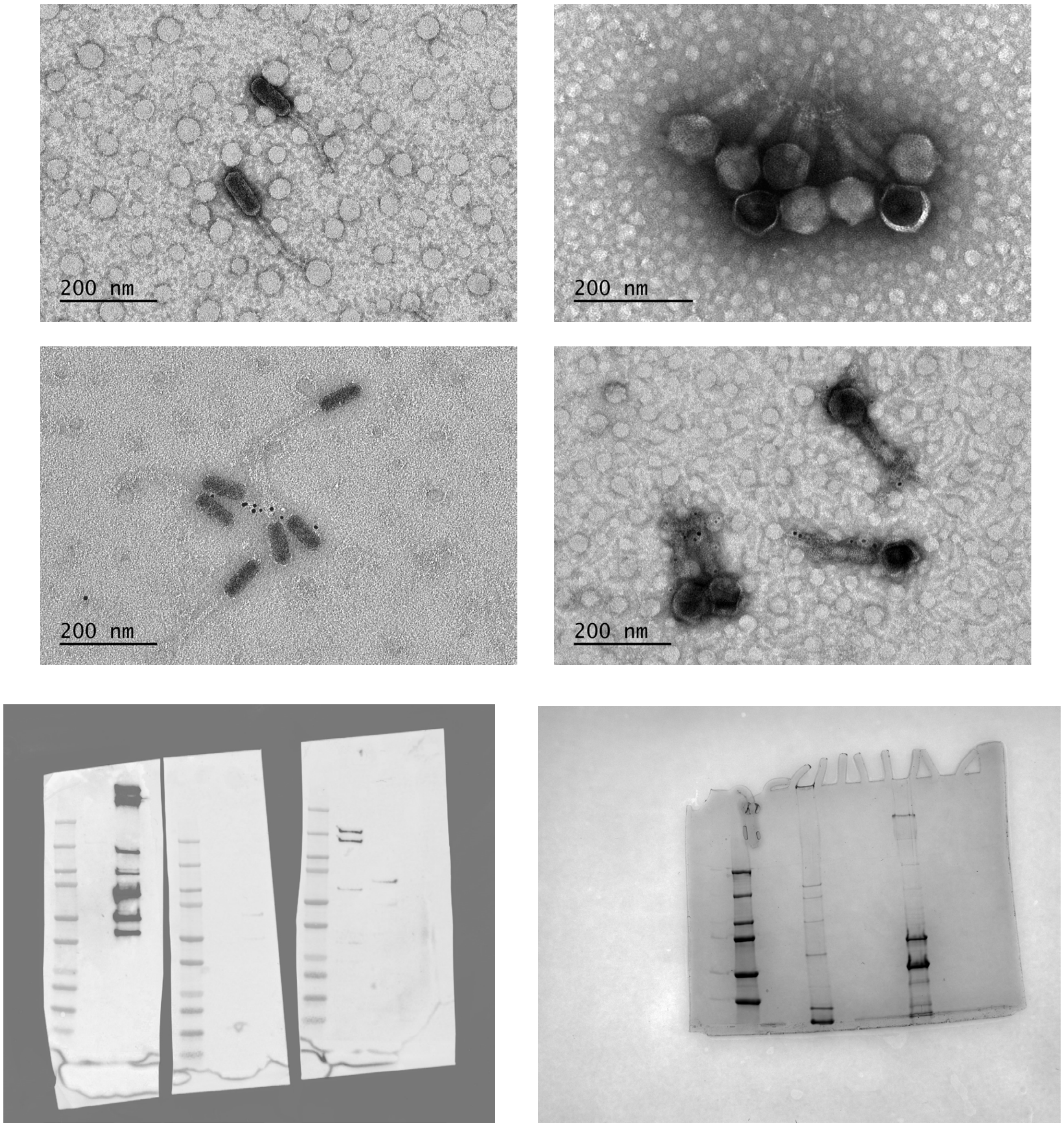

Supplementary figure 6: Raw uncropped, unadjusted images used to generate Figure 4.
