# Supplementary material for: Phage-specific immunity impairs efficacy of bacteriophage targeting Vancomycin Resistant Enterococcus in a murine model
Source: Nat Commun. 2024 Apr 6;15:2993. doi: 10.1038/s41467-024-47192-w (PMC10998888; doi:10.1038/s41467-024-47192-w)
Supplement: Supplementary file 3 — Reporting Summary [file 41467_2024_47192_MOESM3_ESM.pdf]

nature portfolio

Corresponding author(s): Paul Carlson Jr

Last updated by author(s): Feb 14, 2024

Reporting Summary

Nature Portfolio wishes to improve the reproducibility of the work that we publish. This form provides structure for consistency and transparency in reporting. For further information on Nature Portfolio policies, see our Editorial Policies and the Editorial Policy Checklist.

Statistics

For all statistical analyses, confirm that the following items are present in the figure legend, table legend, main text, or Methods section.

n/a	Confirmed	
☐	☒	The exact sample size (*n*) for each experimental group/condition, given as a discrete number and unit of measurement
☐	☒	A statement on whether measurements were taken from distinct samples or whether the same sample was measured repeatedly
☐	☒	The statistical test(s) used AND whether they are one- or two-sided<br>*Only common tests should be described solely by name; describe more complex techniques in the Methods section.*
☐	☒	A description of all covariates tested
☐	☒	A description of any assumptions or corrections, such as tests of normality and adjustment for multiple comparisons
☐	☒	A full description of the statistical parameters including central tendency (e.g. means) or other basic estimates (e.g. regression coefficient) AND variation (e.g. standard deviation) or associated estimates of uncertainty (e.g. confidence intervals)
☐	☒	For null hypothesis testing, the test statistic (e.g. *F*, *t*, *r*) with confidence intervals, effect sizes, degrees of freedom and *P* value noted<br>*Give P values as exact values whenever suitable.*
☒	☐	For Bayesian analysis, information on the choice of priors and Markov chain Monte Carlo settings
☒	☐	For hierarchical and complex designs, identification of the appropriate level for tests and full reporting of outcomes
☒	☐	Estimates of effect sizes (e.g. Cohen's *d*, Pearson's *r*), indicating how they were calculated

Our web collection on statistics for biologists contains articles on many of the points above.

Software and code

Policy information about availability of computer code

Data collection	No software was used for data collection.
Data analysis	GraphPad Prism 5.0 was used to process all data. Phanotate version 1.6.3 was used for analysis of mass spectrometry data.

For manuscripts utilizing custom algorithms or software that are central to the research but not yet described in published literature, software must be made available to editors and reviewers. We strongly encourage code deposition in a community repository (e.g. GitHub). See the Nature Portfolio guidelines for submitting code & software for further information.

Data

Policy information about availability of data

All manuscripts must include a data availability statement. This statement should provide the following information, where applicable:

- Accession codes, unique identifiers, or web links for publicly available datasets
- A description of any restrictions on data availability
- For clinical datasets or third party data, please ensure that the statement adheres to our policy

Source data file is provided as Figures_Raw_Data.xlsx in the supplement.

Research involving human participants, their data, or biological material

Policy information about studies with human participants or human data. See also policy information about sex, gender (identity/presentation), and sexual orientation and race, ethnicity and racism.

Reporting on sex and gender	N/A
Reporting on race, ethnicity, or other socially relevant groupings	N/A
Population characteristics	N/A
Recruitment	N/A
Ethics oversight	N/A

Note that full information on the approval of the study protocol must also be provided in the manuscript.

Field-specific reporting

Please select the one below that is the best fit for your research. If you are not sure, read the appropriate sections before making your selection.

[x] Life sciences [ ] Behavioural & social sciences [ ] Ecological, evolutionary & environmental sciences

For a reference copy of the document with all sections, see nature.com/documents/nr-reporting-summary-flat.pdf

Life sciences study design

All studies must disclose on these points even when the disclosure is negative.

Sample size	For animal studies, sample sizes for the relevant experiment are shown in the figure legend and determined based on the minimal number of animals being sacrificed while fulfilling statistical significance.
Data exclusions	No data were excluded.
Replication	At least three independent biological replicates were performed for all experiments unless otherwise stated.
Randomization	Mice were randomly assigned to treatment groups. No bias in sample allocation was involved.
Blinding	For electron microscopy experiments, the researcher was blinded for immunogold labeling. For other experiments, blinding was not conducted during experiments because reporting for microbial and mouse experiments were quantitative and not subjective based analysis.

Reporting for specific materials, systems and methods

We require information from authors about some types of materials, experimental systems and methods used in many studies. Here, indicate whether each material, system or method listed is relevant to your study. If you are not sure if a list item applies to your research, read the appropriate section before selecting a response.

Materials & experimental systems

n/a	Involved in the study	
[ ]	[x]	Antibodies
[x]	[ ]	Eukaryotic cell lines
[x]	[ ]	Palaeontology and archaeology
[ ]	[x]	Animals and other organisms
[x]	[ ]	Clinical data
[x]	[ ]	Dual use research of concern
[x]	[ ]	Plants

Methods

n/a	Involved in the study	
[x]	[ ]	ChIP-seq
[ ]	[x]	Flow cytometry
[x]	[ ]	MRI-based neuroimaging

Antibodies

Antibodies used	All antibodies used for flow cytometry were commercially available from BD. Each line shows target, fluorophore, clone, and catalog number from BD.<br>CD172a BUV395 P84 740282<br>CD80 BUV737 16-10A1 612773

CD49a BV421 145-2C11 562600
CD64 BV650 X54-5/7.1 740622
NK1.1 BV711 PK136 740663
Siglec F BV786 E50-2440 740956
CD11b AF488 M1/70 557672
Ly6C PerCP-Cy5.5 AL-21 560525
F4/80 PE T45-2342 565410
Ly6G PE-CF594 1A8 562700
CD11c PE-Cy7 HLE 558079
MHC-II AF647 HI30 562367
B220 AF700 RA3-6B2 557957
CD8 APCH7 53-6.7 560182
CD62L BUV395 MEL-14 740218
IgD BUV737 217-170 749300
CD3e BV421 145-2C11 562600
CD69 BV605 H1.2F3 563290
CD273 BV711 TY25 740818
CD19 BV786 1D3 563333
CD138 BB515 281-2 566207
CD44 PerCRP-Cy5.5 IM7 560570
CD73 PE TY/11.8 567215
CD4 PE-CF594 RM4-5 562285
IgM PE-Cy7 R6-60.2 552867
MHC-II AF647 HI30 562367
B220 AF700 RA3-6B2 557957
CD8 APC-H7 53-6.7 560182

Antibodies used as secondary for ELISA:
Goat anti-Mouse IgG (H+L) Secondary Antibody, HRP ThermoFisher cat# 31430
Goat anti-Mouse IgM Secondary Antibody, HRP cat # 31440

Antibody used as secondary for Western Blot:
Goat anti-Mouse IgG, IgM, IgA (H+L) Secondary Antibody, HRP cat #A-10668

Validation

All antibodies were commercially validated for the application used as indicated in the quality assurance literature provided by the supplier.

Animals and other research organisms

Policy information about studies involving animals; ARRIVE guidelines recommended for reporting animal research, and Sex and Gender in Research

Laboratory animals

C57Bl/6 mice were acquired at 5-7 weeks old from Jackson Laboratory (Bar Harbor, ME)

Wild animals

N/A

Reporting on sex

All mice in this study were female.

Field-collected samples

N/A

Ethics oversight

Food and Drug Administration (FDA) Institutional Animal Care and Use Committee (IACUC)

Note that full information on the approval of the study protocol must also be provided in the manuscript.

Plants

Seed stocks

Report on the source of all seed stocks or other plant material used. If applicable, state the seed stock centre and catalogue number. If plant specimens were collected from the field, describe the collection location, date and sampling procedures.

Novel plant genotypes

Describe the methods by which all novel plant genotypes were produced. This includes those generated by transgenic approaches, gene editing, chemical/radiation-based mutagenesis and hybridization. For transgenic lines, describe the transformation method, the number of independent lines analyzed and the generation upon which experiments were performed. For gene-edited lines, describe the editor used, the endogenous sequence targeted for editing, the targeting guide RNA sequence (if applicable) and how the editor was applied.

Authentication

Describe any authentication procedures for each seed stock used or novel genotype generated. Describe any experiments used to assess the effect of a mutation and, where applicable, how potential secondary effects (e.g. second site T-DNA insertions, mosiacism, off-target gene editing) were examined.

Flow Cytometry

Plots

Confirm that:

- [x] The axis labels state the marker and fluorochrome used (e.g. CD4-FITC).
- [x] The axis scales are clearly visible. Include numbers along axes only for bottom left plot of group (a 'group' is an analysis of identical markers).
- [x] All plots are contour plots with outliers or pseudocolor plots.
- [x] A numerical value for number of cells or percentage (with statistics) is provided.

Methodology

Sample preparation	Spleens were extracted and processed into a single-cell suspension by manually filtering tissues through a 70m filter (Corning) into 10 mL of RPMI (Gibco). Cells were centrifuged at 1500 rpm for five minutes and then resuspended in 5mL of Ammonium-Chloride-Potassium (ACK) lysis buffer (Gibco) on ice for 10 minutes. Lysis was stopped with the addition of 10mL RPMI and another centrifugation step.
Instrument	Samples were acquired using the LSRFortessa (BD Biosciences)
Software	Data were analyzed with Flowjo software (version 10.8.1 or higher)
Cell population abundance	For immune analysis in mice, 50,000 total events were were collected.
Gating strategy	Gating strategy is outlined in the supplemental figures. Positive and negative gates were set up using fluorescence minus one (FMO) background intensity controls. Fluorophores were chosen to minimize spectral overlap.

- [x] Tick this box to confirm that a figure exemplifying the gating strategy is provided in the Supplementary Information.
